# The dose-response relationship between sex hormones and hyperuricemia in different gender: NHANES 2013-2016

**DOI:** 10.3389/fendo.2022.1035114

**Published:** 2022-11-01

**Authors:** Guo-yun Li, Xu-dong Qian, Chun-ming Ma, Fu-zai Yin

**Affiliations:** ^1^ Department of Internal Medicine, Hebei Medical University, Shijiazhuang, Hebei, China; ^2^ Department of Endocrinology, The First Hospital of Qinhuangdao, Qinhuangdao, Hebei, China; ^3^ Department of Endocrinology, Affiliated Hospital of Chengde Medical University, Chengde, Hebei, China; ^4^ Department of Neurology, Affiliated Hospital of Chengde Medical University, Chengde, Hebei, China

**Keywords:** hyperuricemia, estradiol, testosterone, NHANES, dose-response

## Abstract

**Objectives:**

To access the dose-response relationship between sex hormones and hyperuricemia (HUA), and to find the cut-off value in different gender.

**Methods:**

9,685 participants were derived from the database of National Health and Nutrition Examination Survey (NHANES). Restricted cubic spline (RCS) analysis were applied to explore the relationship between sex hormones and HUA after adjusting for confounding factors by propensity score match (PSM). Logistic regression was used to estimate the odds ratio (OR) and 95% CI.

**Results:**

The prevalence of HUA was 15.13% in female participants and 22.30% in male participants. Logistic regression analysis showed that estradiol (E2) was independently associated with HUA for a P value of 0.003 and 0.01in female and male participants, respectively. Testosterone (T) was only independently associated with HUA in male participants (P<0.001) but not in female participants (P = 0.59). RCS analysis showed a dose-response relationship between sex hormones and HUA. The risk of HUA increased as E2 lower than 29.6pg/mL in female participants and T lower than 389.1ng/dL in male participants. E2 higher than 23.6pg/ml was an independent risk factor for HUA in male participants.

**Conclusion:**

A dose-response relationship was found between sex hormones and HUA. The cut-off value of E2 in male and female participants was 29.6pg/mL and 23.6pg/mL, respectively, and the cut-off value of T in male participants was 389.1ng/dL. These results provide a reference for preventing HUA and hormone supplement therapy.

## Introduction

The prevalence of hyperuricemia (HUA) in overall population is 13.2% to 20.2% in different countries and regions (1–3), unfortunately, it shows a significant increase in recent years, which has now become another common metabolic disease after diabetes (4–6). HUA is a risk factor for various diseases, including gout, hypertension, diabetes, cardiovascular and cerebrovascular diseases, kidney disease, and metabolic syndrome. It is also an independent risk factor for cardiovascular diseases, diabetes complications, and increased all-cause mortality (7–9). The global burden of disease study find that HUA and gout has brought a substantial economic burden to the world (9). Therefore, it is imperative to explore the risk factors for HUA.

The well-known factors were associated with serum uric acid level include diet, alcohol consumption, obesity, and gene polymorphisms (10, 11). Yet, the relationship between sex hormones and serum uric acid level is complicated. In daily clinical work, we can observe that HUA is more common in men and postmenopausal women. It has been reported that serum uric acid level in postmenopausal women increased by 0.34mg/dl compared with premenopausal women. Serum uric acid levels in men are greater than women by 1mg/dl on average (12). Furthermore, serum uric acid levels are 0.24 mg/dl lower than placebo group in postmenopausal women after hormone supplement therapy at 1-year follow-up (12), suggesting that the serum uric acid level in adults may be related to gonadal hormone concentrations. At present, some studies (13, 14) have shown that the effects of sex hormones on uric acid level differs between women and men. Testosterone (T) is negatively correlated with uric acid levels in male participants and positively associated with uric acid levels in female participants. Likewise, estradiol (E2) is negatively correlated with female uric acid and positively correlated with male uric acid. Other researchers have found that the relationship is no longer significant among postmenopausal women and men over 65, and the mechanism was still unclear (14, 15). At present, there is no objective report on the dose-response relationship between sex hormones and HUA. Therefore, the purpose of this study was to investigate the dose-response relationship between sex hormones and HUA, and to find the cut-off values of E2 and T when the risk of hyperuricemia increased. The results will provide ideas and references for the prevention and treatment of hyperuricemia.

## Materials and methods

### Data source

The NHANES database is a large and representative multi-stage complex sample survey project in the United States. The primary purpose is to understand American citizens’ health and nutrition status. Data were obtained through personal interviews at home, health examinations at mobile examination centers, and laboratory sample analysis. Before completing the NHANES, all participants obtained written informed consent and approved by the National Center of Health Statistics Research Ethics Review Board. The data were de-identified by the National Center for Health Statistics before publication (16). Ethical approval was not required for this study because the study was based on secondary analyses of publicly available data.

**Figure 1:**
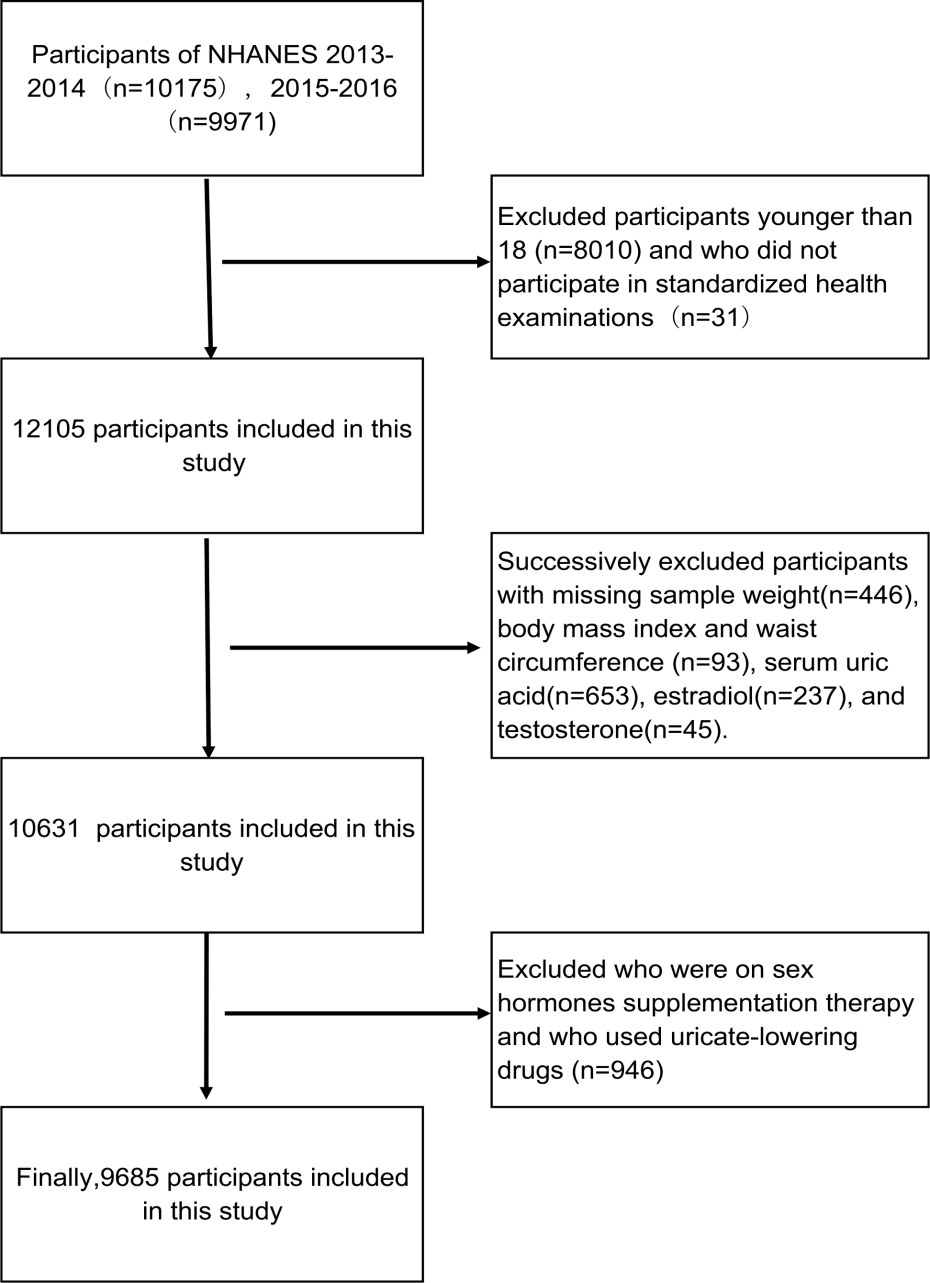
Flow chart of the screening process for the selection of eligible participants.

Participants aged 18-80 years from the database of NHANES 2013-2016 were enrolled in this study. We excluded the participants with missing sample weight, body mass index (BMI), waist circumference (WC), serum uric acid, E2, and T. In addition, individuals who had been treated with anti-hyperuricemia drug and used hormone replacement therapy were excluded. Eventually, 9,685 participants were included in this cross-sectional study (**Figure 1**).

### Variables

Serum uric acid concentrations were measured using the DXC800 and timing endpoint method. The lower limit of uric acid detection was 0.5mg/dL (17), and HUA was defined as uric acid greater than 6mg/dL in female participants and 7mg/dL in male participants. T and E2 were determined by isotope dilution chromatography-tandem mass spectrometry (ID-LC-MS/MS) developed by CDC. The lower limit of T detection was 0.76ng/dL and the lower limit of E2 detection was 2.994pg/mL (17).

### Covariates

Potential confounding factors in this study include age, BMI, WC, ethnicity, alcohol consumption, physical activities (PA) and six diseases. Trained health technicians collect height, weight, and WC measurements at mobile screening centers. BMI is calculated by dividing weight in kilograms by height in meters squared. Alcohol consumption were obtained from alcohol use questionnaire and drinkers were classified into light alcohol consumption (≤2drinks/day) and heavy alcohol consumption (>2 drinks/day). PA were obtained from physical activity questionnaire, total time spent doing activity during a week was evaluated. At least 150min of moderate-intensity or 75min of vigorous-intensity recreational activities per week were used to identify whether adults had adequate physical activity. Six diseases include hypertension (HT), cardiovascular disease (CVD), diabetes mellitus (DM), metabolic syndrome (MS), hyperlipidemia (HLP), and chronic kidney diseases (CKD). HT was defined as any self-reported hypertension, use of an antihypertensive drug, or BP ≥140/90 mmHg. DM was defined as any self-reported diabetes mellitus or use of glucose-lowering drugs or fasting blood glucose ≥7 mmol/L. HLP was defined as any self-reported history or use of lipid-lowering drugs, or serum TC ≥200mg/dL or TG ≥150mg/dL or LDL-C ≥130mg/dL, or HDL-C <40mg/dL in female participants and HDL-C <50mg/dL. MS should meet the diagnostic criteria for MS in ATP or IDF (18, 19). The diagnose of CKD requires an eGFR< 60 mL/min/1.73m^2^ or urinary albumin-to-creatinine ratio (UACR)≥30 mg/g by KDIGO (20). eGFR was calculated using the CKD-EPI equation (21). CVD was determined by the self-reported history of coronary heart disease, congestive heart failure, heart attack, or stroke.

### Statistical analysis

All analyses were conducted using R studio 4.2.1. A two-sided P < 0.05 was considered statistically significant. All data have taken into account the weight of samples. Following the NHANES analysis guidelines, the new sample weight is equal to 1/2 of the weight of the original two years. Continuous variables were presented as mean standard deviation (SD) or median with interquartile range, and categorical variables were presented as frequency and percentages. We used the ANOVA to compare groups of normally distributed parameters and the Kruskal-Wallis test to compare nonparametric variables.

Since the reduction of major hormones in both men and women lead to menopause symptoms, resulting in a significantly higher risk of obesity, hypertension, coronary heart disease and other metabolic diseases, which can significantly affect uric acid levels, such confounding factors may mask the true effect of sex hormones on uric acid, therefore, propensity score matching (PSM) was used to balance the differences in covariates between groups and reduce selection bias. The dependent variable in PSM were grouped on the basis of median E2 (33.1pg/ml) in women and median testosterone (394ng/dl) in men, because it was similar to the sex hormone level when menopausal symptom starts to appear. Six diseases include HT, DM, MS, HLP, CKD and CVD were set as covariate. By calculating the propensity score of samples, the method of caliper matching was used for 1:1 matching, and the matching tolerance was 0.02. Thus, every individual in the low estradiol or testosterone group could be matched with a control individual due to the most similar propensity score.

Our statistical analysis consisted of two steps to investigate the association between sex hormones and HUA. Since E2 and T had a logarithmic relationship with the dependent variable, we took the logarithms of E2 and T and changed them into lge and lgt. In the first step, we analyzed the matched data by multiple logistic regression and establish a fully adjusted regression model. Lge and lgt were independent variables, hyperuricemia was the dependent variable, age, BMI, WC,PA, ethnicity, alcohol consumption and the presence of six diseases including HT, DM, MS, HLP, CKD, and CVD were the covariates. In the second step, based on the results of logistic regression analysis in the first step, RCS model with four nodes (5th, 25th, 65th, and 95th percentiles of T and E2) was used to explore the dose-response relationship between HUA and sex hormone and to determine the cut-off value of E2 and T when the risk of HUA increased significantly. With lge or lgt as the horizontal axis and OR value as the vertical coordinate. OR (95% CI) used RCS adjusting for age, BMI, WC, HT, DM, MS, CKD, CVD, HLP and eth, PA, alcohol drinking. The point on the RCS curve where the OR value was equal to 1 was considered as cut-off value. Nonlinearity was tested using the likelihood ratio test, if P_overall_<0.05 and P_non - linearity_<0.05, indicating a nonlinear dose-response relationship.

## Results

### General characteristics of participants

**Figure 2:**
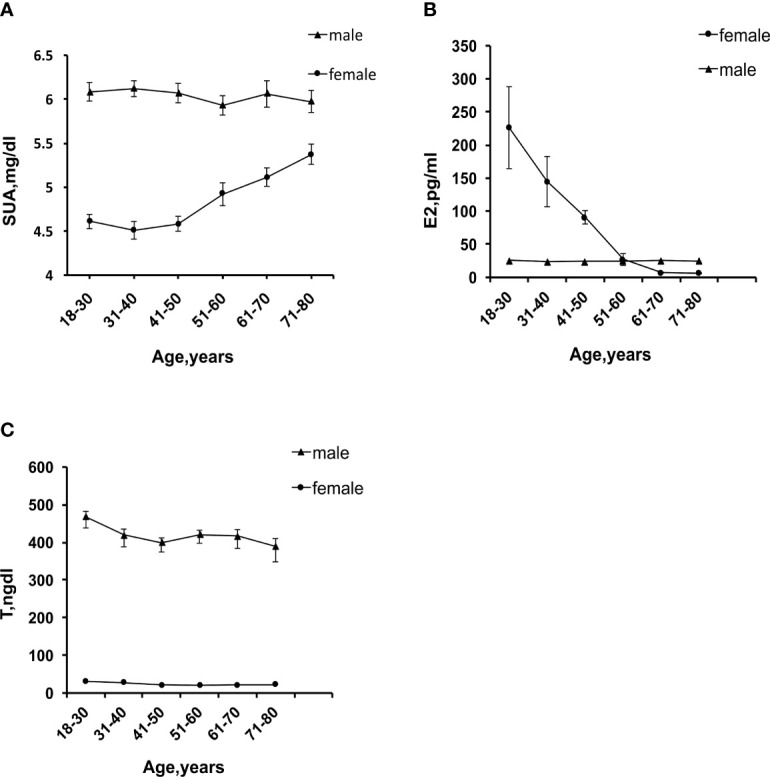
**(A)** Trend chart of serum uric acid levels with age. **(B)** Trend chart of E2 changes with age. **(C)** Trend chart of T changes with age.

A total of 9,685 participants were enrolled in this study, including 4,632 female participants with an average age of 43.66 and 5,053 male participants with an average age of 45.93. The prevalence of HUA was 15.13% in female participants and 22.30% in male participants. As shown in **Tables 1**, **2**, compared with group Q1, the Q4 group had a lower uric acid level, BMI, WC, and incidence of the six diseases. The differences between groups were statistically significant (P<0.001). In female participants, it is noteworthy that the serum uric acid level, BMI, WC, and the incidence of HUA and MS in group Q2 were higher than in group Q1. Line chart of sex hormones and uric acid are shown in **Figure 2**.

**Table 1:** General characteristics of female participants by E2 quartiles.

Characteristics	Q1	Q2	Q3	Q4	P value
Female					
E2, pg/ml	≤ 6.6	6.6-33.1	33.1-93.9	≥93.9	
N	1146 (23.9%)	1177 (24.0%)	1152 (26.0%)	1157 (26.1%)	–
Age, years	57.63 ± 1.66	49.32 ± 1.13	34.41 ± 0.69	34.87 ± 0.72	p < 0.001
UA, mg/dl	4.7 (4.0,5.5)	5.0 (4.2,5.9)	4.6 (3.9,5.3)	4.4 (3.7,5.0)	p < 0.001
BMI, kg/m^2^	26.4 (23.1,30.5)	31.3 (26.4,37.2)	28.7 (23.5,35.6)	26.8 (22.5,32.2)	p < 0.001
WC, cm	93.5 (84.2,103.8)	103.2 (91.8,115)	94.7 (83.2,109.3)	90.7 (80.7,103.6)	p < 0.001
T, ng/dl	14.5 (10.5,21.2)	20.3 (14.7,27.6)	22.8 (16.3,32.3)	26.3 (20.3,35.3)	P < 0.001
HUA, %	17.8 (14.4,21.2)	22.3 (19.9,24.7)	12.3 (10.0,14.6)	8.0 (6.1,9.9)	p < 0.001
HT, %	51.9 (47.6,56.2)	39.1 (35.2,42.9)	17.1 (14.7,19.6)	17.5 (14.8,20.4)	p < 0.001
CKD, %	22.6 (18.9,26.3)	20.7 (17.8,23.6)	10.0 (8.1,11.9)	5.2 (4.2,6.2)	p < 0.001
DM, %	28.0 (25.0,31.0)	26.7 (24.4,29.9)	12.7 (10.6,14.8)	11.1 (8.9,13.4)	p < 0.001
HLP, %	79.3 (76.9,81.8)	73.5 (71.1,75.9)	58.4 (55.1,61.7)	48.9 (44.6,53.3)	p < 0.001
MS, %	12.0 (9.3,14.7)	14.5 (11.7,17.3)	6.3 (4.4,8.1)	5.4 (3.8,7.0)	p < 0.001
CVD, %	12.9 (9.3,14.7)	8.1 (5.6,9.1)	2.1 (1.1,3.1)	1.8 (1.1,2.6)	p < 0.001
eth					
white, %	68.4 (62.8,73.9)	59.8 (53.0,66.6)	55.4 (48.8,62.0)	55.7 (49.3,62.2)	p = 0.889
black, %	9.1 (6.5,11.7)	14.9 (10.5,19.2)	14.0 (10.1,17.9)	14.2 (10.5,17.9)	p = 0.493
Mexican, %	7.1 (4.4, 9.9)	9.5 (6.1,12.8)	12.1 (8.4,15.7)	12.9 (8.7,17.0)	p = 0.687
PA					
low, %	69.1 (64.8,73.5)	68.3 (64.6,71.8)	60.2 (55.6,64.8)	57.3 (53.2,61.3)	p = 0.801
adequate, %	30.9 (26.5,35.3)	31.7 (28.1,35.3)	39.7 (35.1,44.3)	42.6 (38.6,46.7)	p = 0.927
alcohol consumption					
light	68.7 (65.3,72.2)	73.7 (70.3,77.1)	71.2 (68.2,74.1)	73.1 (69.6,76.4)	p = 0.557
heavy	31.2 (27.7,34.7)	26.2 (22.8,29.6)	28.7 (25.8,31.7)	26.9 (23.5,30.3)	p = 0.366

Data are shown as mean with SD or median with interquartile range or n (%) and presented incorporating sample weights or prevalence (%) and 95%CI. E2, estradiol; UA, uric acid; BMI, body mass index; WC, waist circumference; T, testosterone; HUA, hyperuricemia; HT, hypertension; CKD, chronic kidney disease; DM, diabetes mellitus; HLP, hyperlipidemia; MS, metabolic syndrome; CVD, cardiovascular disease; eth, ethnicity; PA, physical activities. Q1 (E2 ≤ 6.6pg/mL), Q2 (6.6< E2 ≤ 33.1pg/mL), Q3 (33.1< E2 <93.9pg/mL), Q4 (E2≥93.9pg/mL).

**Table 2:** General characteristics of male participants by T quartiles.

Characteristics	Q1	Q2	Q3	Q4	P value
Male					
T, ng/dl	≤97.0	97.0-394	394.0-519.5	≥519.5	
N	1270 (24.6%)	1258 (25.1%)	1262 (25.5%)	1263 (24.9%)	–
Age, years	48.67 ± 1.19	47.26 ± 1.01	44.84 ± 1.5	43 ± 1.36	p < 0.001
UA, mg/dl	6.4 (5.5,7.3)	6.1 (5.3,6.9)	5.8 (5.2,6.6)	5.6 (4.9,6.3)	p < 0.001
BMI, kg/m2	31.5 (27.3,36.3)	28.9 (26,32.2)	27.1 (24.4,30.4)	25.1 (22.5,28.4)	p < 0.001
WC, cm	108 (99.4,121.6)	103.2 (94.8,111)	97.4 (88.9,106.5)	91.8 (82.7,101.5)	p < 0.001
E2, pg/ml	19.9 (15.2,25.4)	22.3 (18.1,26.8)	24.4 (19.7,29.6)	28.1 (22.7,34.1)	p < 0.001
HUA, %	35.5 (32.4,38.6)	24.4 (21.1,27.7)	17.6 (14.8,20.3)	12.0 (9.4,14.5)	p < 0.001
HT, %	48.5 (45.4,52.5)	39.4 (35.3,43.5)	33.3 (30.1,36.4)	29.9 (27.3,32.6)	p < 0.001
CKD, %	16.9 (14.5,19.4)	13.0 (10.9,15.1)	8.9 (7.2,10.6)	9.6 (7.3,11.9)	p < 0.001
DM, %	32.6 (30.1,35.2)	24.4 (21.5,27.3)	24.0 (20.6,27.4)	19.8 (16.9,22.8)	p < 0.001
HLP, %	78.8 (75.5,82.2)	70.2 (66.8,73.6)	60.8 (57.4,64.2)	54.7 (51.0,58.4)	p < 0.001
MS, %	19.3 (16.2,22.5)	12.9 (10.8,15.1)	8.7 (6.6,10.8)	5.1 (3.2,7.1)	p < 0.001
CVD, %	11.9 (10.0,13.7)	9.2 (7.1,11.3)	7.2 (5.7,8.7)	7.4 (5.4,9.5)	p < 0.001
eth					
white, %	66.5 (61.1,72.1)	65.7 (60.2,71.2)	66.5 (60.8,72.2)	63.1 (57.8,68.4)	p = 0.485
black, %	8.2 (6.1,10.3)	9.5 (7.1,12.1)	9.8 (7.5,12.1)	12.5 (9.3,15.8)	p = 0.515
Mexican, %	10.2 (6.8,13.6)	10.2 (6.9,13.5)	9.7 (6.4,13.1)	9.3 (6.4,12.3)	p = 0.695
PA					
low, %	63.1 (59.3,66.9)	59.8 (56.7,63.0)	55.9 (52.2,59.5)	51.6 (47.4,55.8)	p = 0.295
adequate, %	36.8 (33.1,40.6)	40.1 (36.9,43.3)	44.1 (40.4,47.7)	48.3 (44.1,52.5)	p = 0.802
alcohol consumption					
light	69.5 (66.6,72.4)	72.5 (69.2,75.8)	74.9 (72.2,77.5)	74.0 (70.2,77.7)	p = 0.539
heavy	30.4 (27.6,33.3)	27.4 (24.1,30.7)	25.1 (22.4,27.7)	25.9 (22.2,29.7)	p = 0.678

Data are shown as mean with SD or median with interquartile range or n (%) and presented incorporating sample weights or prevalence (%) and 95%CI. E2, estradiol; UA, uric acid; BMI, body mass index; WC, waist circumference; T, testosterone; HUA, hyperuricemia; HT, hypertension; CKD, chronic kidney disease; DM, diabetes mellitus; HLP, hyperlipidemia; MS, metabolic syndrome; CVD, cardiovascular disease; eth, ethnicity; PA, physical activities. Q1 (T ≤ 97.0ng/dL), Q2 (97.0< T ≤394ng/dL), Q3 (394.0< T <519.5ng/dL), Q4 (T ≥519.5ng/dL).

### Associations between sex hormones and HUA

Before PSM, the incidence of six diseases was significantly higher in the low sex hormone levels group than in the high levels group (*P* = < 0.001). After 1:1 PSM, 2,868 female participants and 4,030 male participants were enrolled in the analysis. There were no significant differences in the incidence of six diseases between the two groups (**Table 3**).

**Table 3:** |Baseline comparison of influencing factors before and after PSM.

Characteristics	Pre-PSM			After-PSM		
Female						
E2, pg/ml	≤33.1	>33.1	P value	≤33.1	>33.1	P value
N	2323 (47.87%)	2309 (52.13%)	–	1434 (49.74%)	1434 (50.26%)	–
HT, %	46.5 (42.3,48.6)	17.52 (15.7,19.0)	p < 0.001	24.4 (22.5,27.0)	26.9 (24.2,29.9)	p = 0.154
CKD, %	21.94 (18.9,24.5)	8 (6.5,8.7)	p < 0.001	12.5 (10.1,15.2)	11.3 (9.6,12.8)	p = 0.381
DM, %	27.9 (24.8,29.8)	11.9 (10.3,13.5)	p < 0.001	14.4 (12.6,16.4)	16.2 (14.0,18.3)	p = 0.278
HLP, %	76.8 (74.8,78.1)	54.2 (51.4,56.0)	p < 0.001	68.2 (65.5,71.3)	65.8 (62.6,68.7)	p = 0.272
MS, %	13.6 (11.4,15.2)	5.8 (4.7,7.0)	p < 0.001	8.1 (5.9,10.2)	7.7 (6.2,9.4)	p = 0.840
CVD, %	10.1 (8.0,11.4)	2.0 (1.3,2.7)	p < 0.001	2.8 (1.8,3.7)	3.2 (2.1,4.2)	p = 0.507
Male						
T, ng/dl	≤394	>394		≤394	>394	
N	2528 (49.65%)	2525 (50.35%)		2015 (49.75%)	2015 (50.25%)	
HT, %	43.8 (41.1,46.7)	31.6 (29.9,33.3)	p < 0.001	36.2 (33.2,39.2)	36.3 (34.1,38.5)	p = 0.948
CKD, %	14.9 (13.2,16.7)	9.2 (7.9,10.5)	p < 0.001	9.7 (8.1,10.9)	10.5 (8.8,11.9)	p = 0.213
DM, %	28.7 (27.2,30.0)	21.9 (19.9,23.9)	p < 0.001	23.6 (21.2,26.7)	25.4 (22.9,27.8)	P = 0.255
HLP, %	74.5 (71.9,76.9)	57.8 (55.2,60.4)	p < 0.001	68.9 (66.5,72.6)	69.2 (66.0,70.3)	p = 0.921
MS, %	16.1 (14.3,17.8)	6.9 (5.6,8.3)	p < 0.001	9.6 (7.7,11.4)	8.5 (6.9,10.2)	P = 0.423
CVD, %	10.5 (9.0,12.0)	7.3 (6.0,8.6)	p = 0.001	8.1 (6.1,9.7)	7.1 (5.9,8.8)	P = 0.119

Data are shown as n (%) and presented incorporating sample weights or prevalence (%) and 95%CI. E2, estradiol; UA, uric acid; BMI, body mass index; WC, waist circumference; T, testosterone; HUA, hyperuricemia; HT, hypertension; CKD, chronic kidney disease; DM, diabetes mellitus; HLP, hyperlipidemia; MS, metabolic syndrome; CVD, cardiovascular disease.

**Figure 3:**
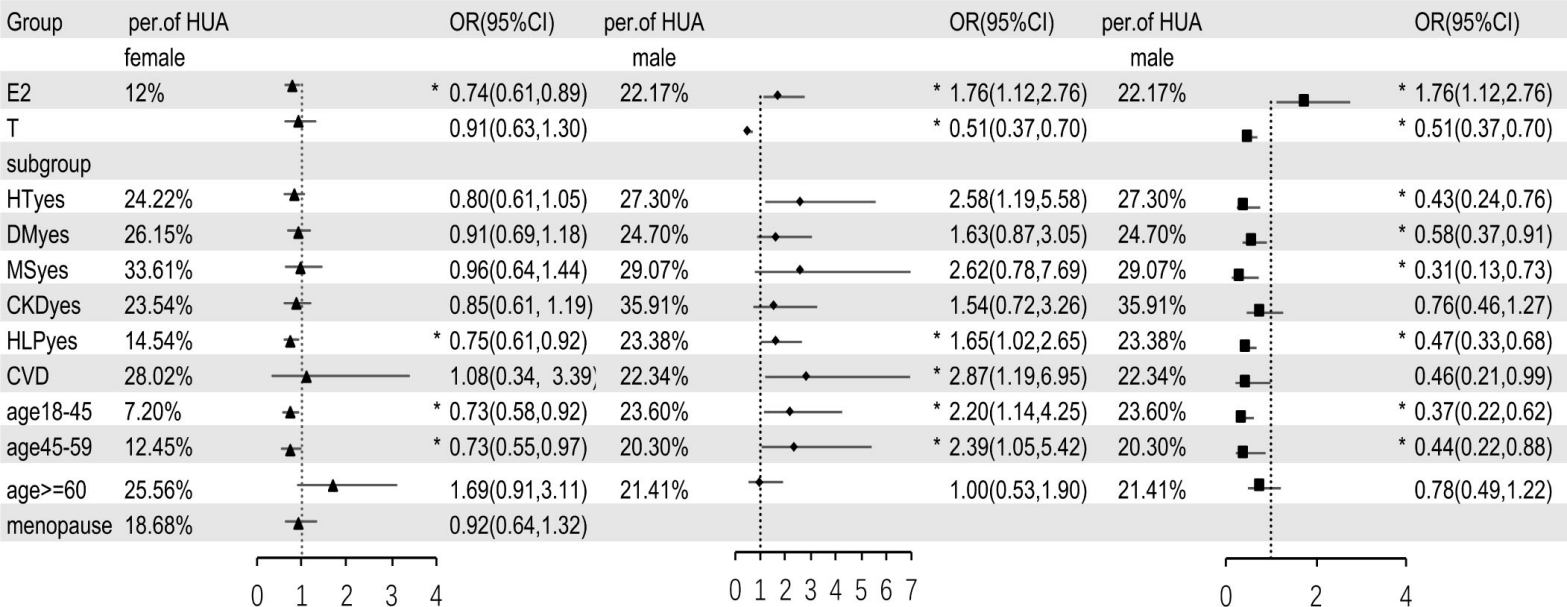
Multivariable logistic regression analysis between sex hormones and HUA, after adjusting for variables (except for the grouped variables) such as age, BMI, WC, HT, DM, MS, CKD, CVD, HLP, eth, PA and alcohol drinking. E2, estradiol; T, testosterone; HT, hypertension; DM, diabetes mellitus; MS, metabolic syndrome; CKD, chronic kidney disease; HLP, hyperlipidemia; CVD, cardiovascular disease; eth, ethnic; PA, physical activities; per of HUA, percentage of hyperuricemia; HT/DM/MS/CKD/HLP/CVDyes means people who have a history of hypertension/diabetes mellitus/metabolic syndrome/chronic kidney disease/hyperlipidemia/cardiovascular disease. In subgroup analysis, ▲ means estradiol in female, ◆ means estradiol in male, ■ means testosterone in male. * means P < 0.05.

We analyzed the matching data by fully adjusted multivariable logistic regression model (**Figure 3**). The results showed that, in female participants, E2 was a protective factor for HUA, and the prevalence of HUA decreased by 26% for every log-unit increase of E2 (OR=0.74,95%CI: 0.61-0.89, P = 0.003). Additionally, we found T was not independently associated with HUA (OR=0.91,95%CI: 0.63-1.30, P = 0.59). In the subgroup analyses, the association between E2 and HUA disappeared when the participants were older than 60 years, menopausal, or combined with HT, DM, MS, CKD, and CVD; E2 was still an independent protective factor for HUA when participants were less than 60 years or combined with HLP. In male participants, E2 was an independent risk factor for HUA, and with every log-unit increase of E2, the prevalence of HUA increased by 76% (OR=1.76,95%CI:1.12-2.76, P = 0.01). T was identified as a protective factor for HUA and with every log-unit increase of T, the prevalence of HUA decreased by 49% the prevalence of HUA (OR=0.51,95%CI:0.37-0.70, P< 0.001). Subgroup analysis showed no independent correlation between E2 and HUA in patients with HT, DM, MS, CKD, or age≥60 years; E2 was still an independent risk factor for HUA when participants were less than 60 years, or combined with HLP or CVD. Furthermore, there was no correlation between T and HUA in patients with CKD, CVD, or age≥60 years; T was still an independent protective factor for HUA when participants were less than 60 years or combined with HT, DM, MS or HLP.

### Dose-response relationship between sex hormones and HUA

**Figure 4:**
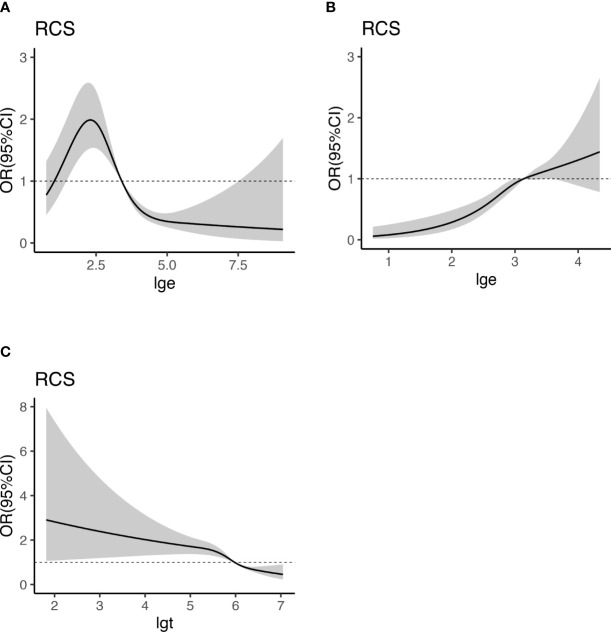
Dose-response relationship between E2, T, and HUA. RCS analysis shows a dose-response relationship between HUA and E2 in female **(A)**, E2 in male **(B)**, and T in male **(C)**. OR were adjusted for age, BMI, WC, HT, DM, MS, CKD, CVD, HLP and eth, PA, alcohol drinking. E2, estradiol; UA, uric acid; BMI, body mass index; WC, waist circumference; T, testosterone; HUA, hyperuricemia; HT, hypertension; CKD, chronic kidney disease; DM, diabetes mellitus; HLP, hyperlipidemia; MS, metabolic syndrome; CVD, cardiovascular disease; eth, ethnic; PA, physical activities; lge, logarithms of E2; lgt, logarithms of T.

We embedded the statistically significant results in multivariable logistic regression analysis into the RCS model with 4 nodes (5th, 25th, 65th, and 95th percentiles of T and E2) to analyze the relationship between E2, T, and HUA. Since E2 and T have a logarithmic relationship with the dependent variable, we take the logarithms of E2 and T and change them into lge and lgt. The RCS curve was drawn with lge or lgt as the abscissa and OR value as the ordinate (**Figure 4**), and the shadow represents 95%CI. OR (95% CI) used RCS adjusting for age, BMI, WC, HT, DM, MS, CKD, CVD, HLP and eth, PA, alcohol drinking. As shown in **Figure 4**, E2 and HUA presented a nonlinear dose-response relationship in female participants (P_overall_<0.001, P_non-linearity_<0.001). In male participants, E2 and HUA showed a linear dose-response relationship (P_overall_<0.001, P_non-linearity_ =0.2262), and T and HUA have a non-linear dose-response relationship (P_overall_<0.001, P_non - linearity_<0.001). In female participants, the risk of HUA was highest when E2 was 10.90pg/mL (lge=2.38, OR=1.97,95%CI:1.53-2.53).29.6pg/mL (lge=3.38, OR=1.01,95%CI: 1.01-1.02) could be regarded as the cut-off point. In male, the cut-off point of E2 was 23.6pg/mL (lge=3.16, OR=1,95%CI:1.00-1.31) and T cut-off point was 389.1ng/dl (lgt=5.96, OR=1,95%CI:1.00-1.31)

## Discussion

In this study, the prevalence of HUA in male and female participants was 15.13% and 22.30%, respectively, which was consistent with previous reports. After adjusting for confounding factors, the results of multivariable regression analysis confirmed that E2 and T were independently associated with HUA in male participants, but in female participants, only E2 was independently associated with HUA. RCS analysis showed that, in female participants, E2 had a nonlinear dose-response relationship with HUA, in male participants, E2 had a linear dose-response relationship with HUA, while the T had a nonlinear dose-response relationship with HUA. Yutang Wang’s study found that E2 is positively correlated with serum uric acid in male participants (β=0.079, P<0.001) and negatively correlated with the uric acid level in female participants (β=-0.05, P=0.002) (13). Other scholars (14, 15) conducted subgroup analyses and found that the relationship between E2, T, and HUA disappeared in postmenopausal women and men over 65. The above results were consistent with the results of this study. In addition, the relationship between sex hormones and HUA was also not apparent in patients combined with DM, CKD, MS, and CVD. It can be seen that the above diseases may affect serum uric acid levels and thus conceal the effect of sex hormones on uric acid, which confirms the research results of above scholars to some extent (22, 23).

The intersection of the dotted line and RCS curve in **Figure 4** (OR=1) was set as the critical point. It was considered that, in female participants, E2 greater than 29.6pg/mL (lge = 3.38) was a protective factor for HUA and that lower than 29.6pg/mL was a risk factor. In male participants, E2 higher than 23.6pg/mL(lge=3.16) was a risk factor for HUA, while E2 lower than 23.6pg/mL ((lge=3.16) was not independently associated with HUA. T was a protective factor for HUA when it was higher than 389.1ng/dL (lgt=5.96) in men, whereas it was a risk factor for HUA. This provides a reference for the prevention and hormone supplement therapy of HUA.

The Endocrine Society of America (24) recommends hormone supplementation for symptomatic postmenopausal women, and women within 10 years of menopause or younger than 60 are most likely to benefit from treatment (25). A Chinese study (26) investigated 424 perimenopause women aged 45-55 in sever major cities. The results showed that the average estradiol level of premenopausal women was 67.30pg/ml, and the estrogen level decreased to 35.14pg/ml within one year of menopause. Five years after menopause, woman’s estradiol level drops to 21.25pg/ml. Symptoms of menopausal syndrome include vasomotor symptoms, genitourinary, cardiovascular, osteoarticular and autonomic nervous systems and nervous system symptoms (27, 28). Cheng Fangping’s study (26) showed that the severity of menopausal symptoms gradually increased in the first two years of menopause, alleviated in the first two to five years of menopause, but increased again after five years of menopause. The Owens study (29) of 521 women aged 42 to 50, followed for 4 years, found that menopausal symptoms gradually worsened after 1 year of menopause. Our cut-off value was 29.6pg/ml, which was approximately equivalent to the estradiol level about 1 year after menopause. The risk of HUA increases gradually as E2 less than 29.6pg/ml. This cut-off point was similar to above thresholds According to our results, we suggested doctor should monitor the E2 level and make it reach more than 29.6 pg/mL during hormone replacement therapy, thereby reducing the risk of HUA. We also have reason to speculate when E2 levels are above 29.6 pg/mL, it can better prevent osteoporosis, improve lipid metabolism, prevent cardiovascular and cerebrovascular diseases. Of course, this needs to be confirmed in subsequent studies.

The prevalence of low testosterone (total T<11.1nmol/L) in men over 60 is about 20% (30), and low T is associated with insulin resistance, hypertension, and CVD (31, 32). The Endocrine Society guidelines suggested that clinicians could prescribe testosterone supplementation therapy for men older than 65 after an explicit discussion of the risks and benefits (33), However, different associations disagree with the total testosterone threshold for supplementation therapy (34). Loss of libido and vigour start to appear when T falls below 15nmol/L (432ng/dL) and certainly below 12nmol/L (349ng/dL) when obesity also emerges. Depressive mood, lack of concentration and sleep disturbances occur at levels below 10 nmol/L (288ng/dL) (35). The cut-off point of this study was 389.1ng/dL. which was similar to the above value. Therefore, we recommend that T falls below 389.1ng/dL, attention should be paid to the occurrence of HUA, which was similar to the above cut-off point.

In actually, lower serum uric acid levels are not better. The antioxidant effect of uric acid is protective on the nervous system, intestinal tract, and kidney (36–38). Some researchers have demonstrated that low uric acid levels are associated with an increased risk of mortality in patients (39, 40). Therefore, decreased sex hormones levels and increased uric acid levels in the elderly may be a protective mechanism that needs further exploration in subsequent studies.

The data in this study came from an extensive sample sampling survey and were all weighted. The influence of confounding factors was adjusted twice in the statistical process, so the results were reliable. However, there were still some noted limitations. 1. The research data came from the NHANES database, in which part of the home interviews was conducted by questionnaire survey, and some participants may have poor compliance. 2. This study was retrospective, and although most potential confounding factors were adjusted in the multivariable analysis, other factors, such as dietary factors, may influence the results. 3. Normal levels of sex hormones fluctuate in cycles, so the measurement timing may affect the results.

## Data availability statement

The raw data supporting the conclusions of this article will be made available by the authors, without undue reservation.

## Author contributions

G-YL analyzed the data and wrote the paper. X-DQ and C-MM: participated in its design and coordination. F-ZY conceived of this study. All authors contributed to the article and approved the submitted version.

## Funding

This study was supported by natural Science foundation of Hebei province (grant no. 20377708D). The funders had no role in the study design, data collection and analysis, decision to publish, or preparation of the manuscript.

## Acknowledgments

The authors thank AiMi Academic Services (www.aimieditor.com) for the English language editing and review services. Thanks to Zhangjing (Shanghai Tongren Hospital) and his nhanesR package.

## Conflict of interest

The authors declare that the research was conducted in the absence of any commercial or financial relationships that could be construed as a potential conflict of interest.

## Publisher’s note

All claims expressed in this article are solely those of the authors and do not necessarily represent those of their affiliated organizations, or those of the publisher, the editors and the reviewers. Any product that may be evaluated in this article, or claim that may be made by its manufacturer, is not guaranteed or endorsed by the publisher.
